# Supplementary material for: Seed priming enhances seed germination and plant growth in four neglected cultivars of Capsicum annuum L
Source: PeerJ. 2024 Oct 28;12:e18293. doi: 10.7717/peerj.18293 (PMC11526797; doi:10.7717/peerj.18293)
Supplement: Supplemental Information 1 — Pepper seeds (cut in half) stained by tetrazolium solution: a,b) Control seeds, a) viable and vigorous, b) viable non-vigorous; c,d) Priming with KNO3 6% 96h , c) viable and vigorous, d) viable non-vigorous; e,f)Acid priming with HCl at two different times, e) 5’ non-viable; f)10’ non-viable. [file peerj-12-18293-s001.pdf]

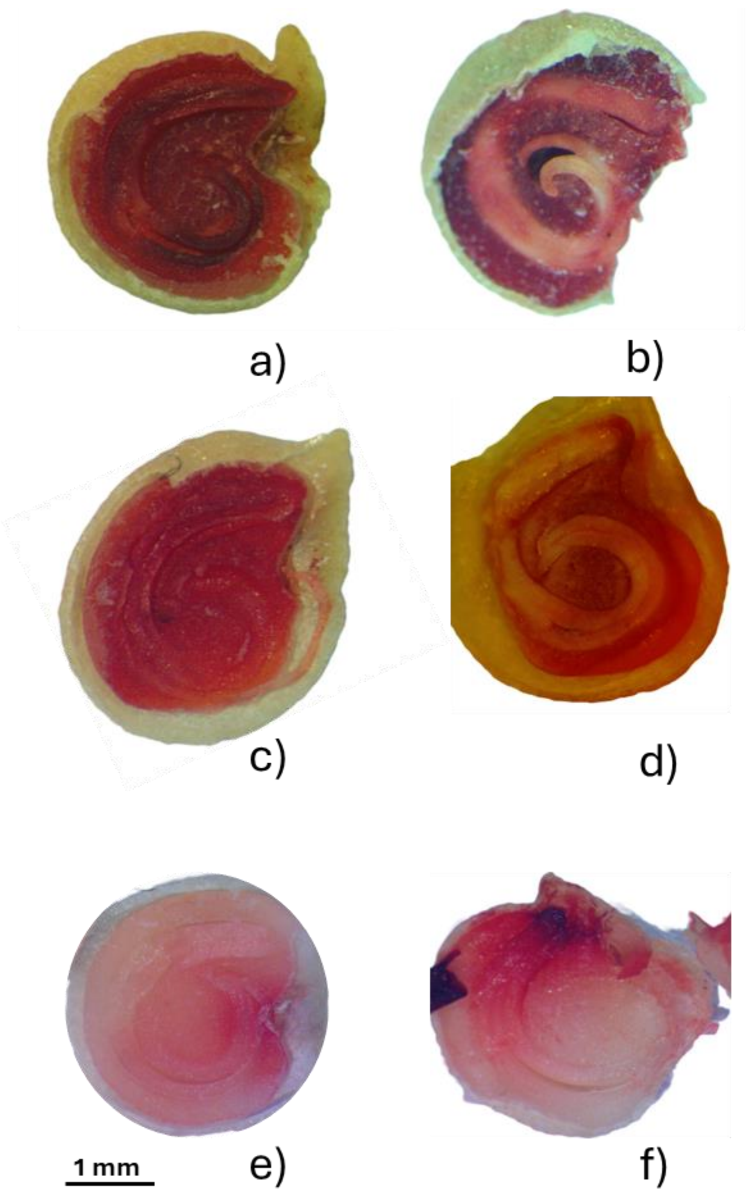

Figure S1. Pepper seeds (cut in half) stained by tetrazolium solution: *a,b*) Control seeds, *a)* viable and vigorous, *b)* viable non-vigorous; *c,d*) Priming with KNO_3 6% 96h , *c)* viable and vigorous, *d)* viable non-vigorous; *e,f*)Acid priming with HCl at two different times, *e)* 5’ non-viable; *f)*10’ non-viable.
