# Supplementary material for: Seed priming enhances seed germination and plant growth in four neglected cultivars of Capsicum annuum L
Source: PeerJ. 2024 Oct 28;12:e18293. doi: 10.7717/peerj.18293 (PMC11526797; doi:10.7717/peerj.18293)
Supplement: Supplemental Information 2 — MGT, morphological data, SVI, DGI and T50. P-values < 0.05 are reported in italic. [file peerj-12-18293-s002.docx]

| **Table S1.** Summary of the general linear models (GLMs) testing for main and interactive effects of cultivar (*CV*), Treatments and Time for the viables: MGT, morphological data, SVI, DGI and T50. **P-values** < 0.05 are reported in italic. | | | | | | | | | | | | | | | |  |
| --- | --- | --- | --- | --- | --- | --- | --- | --- | --- | --- | --- | --- | --- | --- | --- | --- |
|  |  |  |  |  |  |  |  |  |  |  |  |  |  |  |  |  |
| **Effect** | **d.f.** | **SS** | **MS** | **F** | **p** |  | **SS** | **MS** | **F** | **p** |  | **SS** | **MS** | **F** | **p** |  |
|  |  | **MGT** |  |  |  |  | **Shoot weight** |  |  |  |  | **Root weight** |  |  |  |  |
| *CV* | 3 | 36.44 | 12.15 | 59.9 | *<0.001* |  | 0.73 | 0.24 | 9.09 | *<0.001* |  | 0.40 | 0.13 | 19.70 | *<0.001* |  |
| Treatment | 4 | 383.62 | 95.90 | 473.3 | *<0.001* |  | 10.18 | 2.54 | 94.96 | *<0.001* |  | 3.90 | 0.97 | 145.59 | *<0.001* |  |
| Time | 2 | 15.85 | 7.93 | 39.1 | *<0.001* |  | 0.29 | 0.15 | 5.43 | *<0.001* |  | 0.12 | 0.06 | 9.13 | *<0.001* |  |
| *CV**Treatment | 12 | 53.90 | 4.49 | 22.2 | *<0.001* |  | 4.31 | 0.36 | 13.41 | *<0.001* |  | 1.14 | 0.10 | 14.25 | *<0.001* |  |
| *CV**Time | 6 | 9.60 | 1.60 | 7.9 | *<0.001* |  | 0.21 | 0.03 | 1.30 | 0.26 |  | 0.11 | 0.02 | 2.81 | *0.01* |  |
| Treatment*Time | 8 | 37.74 | 4.72 | 23.3 | *<0.001* |  | 2.03 | 0.25 | 9.47 | *<0.001* |  | 1.14 | 0.14 | 21.21 | *<0.001* |  |
| *CV**Treatment*Time | 24 | 8.69 | 0.36 | 1.8 | *0.02* |  | 3.45 | 0.14 | 5.37 | *<0.001* |  | 1.93 | 0.08 | 12.02 | *<0.001* |  |
| **Effect** | **d.f.** | **SS** | **MS** | **F** | **p** |  | **SS** | **MS** | **F** | **p** |  | **SS** | **MS** | **F** | **p** |  |
|  |  | **Tot weight** |  |  |  |  | **Root length** | |  |  |  | **Shoot length** | |  |  |  |
| *CV* | 3 | 3.53 | 1.18 | 26.23 | *<0.001* |  | 250.09 | 83.36 | 23.84 | *<0.001* |  | 1.33 | 0.44 | 0.78 | 0.50 |  |
| Treatment | 4 | 30.42 | 7.61 | 169.39 | *<0.001* |  | 1147.66 | 286.91 | 82.06 | *<0.001* |  | 126.75 | 31.69 | 56.15 | *<0.001* |  |
| Time | 2 | 0.15 | 0.08 | 1.69 | 0.19 |  | 62.94 | 31.47 | 9.00 | *<0.001* |  | 5.11 | 2.55 | 4.52 | *0.01* |  |
| *CV**Treatment | 12 | 11.12 | 0.93 | 20.64 | *<0.001* |  | 301.22 | 25.10 | 7.18 | *<0.001* |  | 22.39 | 1.87 | 3.31 | *<0.001* |  |
| *CV**Time | 6 | 0.74 | 0.12 | 2.75 | *0.01* |  | 368.22 | 61.37 | 17.55 | *<0.001* |  | 4.81 | 0.80 | 1.42 | 0.21 |  |
| Treatment*Time | 8 | 5.78 | 0.72 | 16.09 | *<0.001* |  | 930.21 | 116.28 | 33.26 | *<0.001* |  | 64.40 | 8.05 | 14.26 | *<0.001* |  |
| *CV**Treatment*Time | 24 | 9.83 | 0.41 | 9.12 | *<0.001* |  | 1123.21 | 46.80 | 13.39 | *<0.001* |  | 54.15 | 2.26 | 4.00 | *<0.001* |  |
| **Effect** | **d.f.** | **SS** | **MS** | **F** | **p** |  | **SS** | **MS** | **F** | **p** |  | **SS** | **MS** | **F** | **p** |  |
|  |  | **SVI** |  |  |  |  | **DGI** |  |  |  |  | **T50** |  |  |  |  |
| *CV* | 3 | 22452 | 7484 | 18 | *<0.001* |  | 28769 | 9590 | 22 | *<0.001* |  | 159 | 53 | 4068 | *<0.001* |  |
| Treatment | 4 | 243380 | 60845 | 144 | *<0.001* |  | 93674 | 23419 | 54 | *<0.001* |  | 1070 | 267 | 20500 | *<0.001* |  |
| Time | 2 | 10392 | 5196 | 12 | *<0.001* |  | 3765 | 1883 | 4 | *0.01* |  | 73 | 36 | 2783 | *<0.001* |  |
| *CV**Treatment | 12 | 78711 | 6559 | 16 | *<0.001* |  | 18269 | 1522 | 4 | *<0.001* |  | 271 | 23 | 1732 | *<0.001* |  |
| *CV**Time | 6 | 3744 | 624 | 1 | 0.19 |  | 28523 | 4754 | 11 | *<0.001* |  | 12 | 2 | 149 | *<0.001* |  |
| Treatment*Time | 8 | 74225 | 9278 | 22 | *<0.001* |  | 86600 | 10825 | 25 | *<0.001* |  | 241 | 30 | 2305 | *<0.001* |  |
| *CV**Treatment*Time | 24 | 73275 | 3053 | 7 | *<0.001* |  | 104438 | 4352 | 10 | *<0.001* |  | 154 | 6 | 493 | *<0.001* |  |
